# The secretion and biological function of tumor suppressor maspin as an exosome cargo protein

**DOI:** 10.18632/oncotarget.13302

**Published:** 2016-11-11

**Authors:** Ivory Dean, Sijana H Dzinic, M. Margarida Bernardo, Yi Zou, Vickie Kimler, Xiaohua Li, Alexander Kaplun, James Granneman, Guangzhao Mao, Shijie Sheng

**Affiliations:** ^1^ Department of Pathology, Wayne State University School of Medicine, MI, USA; ^2^ Department of Oncology, Wayne State University School of Medicine, MI, USA; ^3^ The Tumor Biology and Microenvironment Program, Karmanos Cancer Institute, MI, USA; ^4^ Department of Psychiatry and Behavioral Neurosciences, Wayne State University School of Medicine, MI, USA; ^5^ Department of Chemical Engineering and Materials Science, Wayne State University, MI, USA; ^6^ Center for Bioengineering and Tissue Regeneration, The University of California San Francisco, San Francisco, CA, USA; ^7^ Ocular Structure and Imaging Facility, Eye Research Institute, Oakland University, Rochester Hills, MI, USA; ^8^ Zhangjiagang Aoyang Hospital, Nanjing Medical University, Jiangsu, China; ^9^ Variantyx, Framingham, MA, USA

**Keywords:** exosome, tumor progression, exosome cargo, tumor microenvironment, electron microscopy

## Abstract

Maspin is an epithelial-specific tumor suppressor shown to exert its biological effects as an intracellular, cell membrane-associated, and secreted free molecule. A recent study suggests that upon DNA-damaging g-irradiation, tumor cells can secrete maspin as an exosome-associated protein. To date, the biological significance of exosomal secretion of maspin is unknown. The current study aims at addressing whether maspin is spontaneously secreted as an exosomal protein to regulate tumor/stromal interactions. We prepared exosomes along with cell extracts and vesicle-depleted conditioned media (VDCM) from normal epithelial (CRL2221, MCF-10A and BEAS-2B) and cancer (LNCaP, PC3 and SUM149) cell lines. Atomic force microscopy and dynamic light scattering analysis revealed similar size distribution patterns and surface zeta potentials between the normal cells-derived and tumor cells-derived exosomes. Electron microscopy revealed that maspin was encapsulated by the exosomal membrane as a cargo protein. While western blotting revealed that the level of exosomal maspin from tumor cell lines was disproportionally lower relative to the levels of corresponding intracellular and VDCM maspin, as compared to that from normal cell lines, maspin knockdown in MCF-10A cells led to maspin-devoid exosomes, which exhibited significantly reduced suppressive effects on the chemotaxis activity of recipient NIH3T3 fibroblast cells. These data are the first to demonstrate the potential of maspin delivered by exosomes to block tumor-induced stromal response, and support the clinical application of exosomal maspin in cancer diagnosis and treatment.

## INTRODUCTION

Maspin is a 42 kDa epithelial-specific tumor suppressor that predicts a better cancer prognosis, and is down-regulated in the progression of breast, prostate, lung, and esophageal squamous cancers [1–4]. Consistently, maspin has been shown to restrict tumor cell stemness [5] by preserving the epigenetic program for differentiation [6], and to inhibit tumor invasion and metastasis, at least in part, by blocking tumor-induced extracellular matrix remodeling [7]. Further, accumulated evidence showed that maspin expression in epithelial cells plays a critical role in suppressing stromal activities. For example, maspin expression inhibits tumor angiogenesis [8–10], tumor-induced bone remodeling [8], and stimulates anti-tumor immune response [11].

The maspin protein sequence aligns with members of the serine protease inhibitor (serpin) superfamily [12]. Maspin is one of the most ancient members of the serpin superfamily, with several unique sequence and conformational features [13]. Based on the X-ray crystallographic analysis [14, 15], maspin protein can spontaneously switch between an open and a closed conformation in the G-helix, which could be involved in the dynamics of its protein-protein interactions. The reactive center loop (RCL) of maspin has a sequence that prevents its insertion into the β-sheets, which renders maspin non-inhibitory against an active serine protease. Instead, maspin acts as a serpin-like molecule to cross-inhibit serine protease-like pro-urokinase plasminogen activator (pro-uPA) and histone deacetylase 1 (HDAC1) [16]. Maspin does not have an apparent signature sequence to direct its trafficking to specific subcellular compartments. While it is predominantly localized within the cell, in the nuclei and the cytoplasm, it can also be cell-surface associated, and secreted to the medium or extracellular milieu [2, 3, 17–20]. As revealed by our earlier studies, maspin in different subcellular localizations may have different molecular targets [21–23]. For example, nuclear maspin inhibits HDAC1 and controls the expression of a small set of genes critical for epithelial differentiation [6] while extracellular maspin inhibits cell surface-associated pro-uPA [24]. Interestingly, Yu *et al*. showed that upon DNA-damaging irradiation, non-small cell lung cancer H460 cells secrete maspin as an exosomal protein in a p53-dependent manner [25].

Exosomes are 30–150 nm cholesterol- and sphingomyelin-rich bilayer vesicles of endosome origin, containing proteins, messenger RNAs and micro RNAs [26–29]. The genesis and secretion of exosomes *via* the invagination of limiting multivesicular body (MVB) membrane requires the endosomal sorting complex (ESCRT) which consists of two hallmark exosome cargo proteins: programmed cell death 6 interacting protein (Alix) and tumor susceptibility gene 101 protein (Tsg101) [30]. Accumulated evidence suggests that exosomes function as signalosomes for several biological processes, including antigen presentation and delivery of transcription factors and infectious particles into recipient cells [31]. Cancer-derived exosomes have been shown to promote tumor progression, enhance endothelial cell migration and angiogenesis, and promote tumor evasion of immune surveillance [32–37].

Our study is the first to demonstrate that maspin is naturally secreted *via* the exosomal pathway in normal and cancer cell lines regardless of p53 status and that maspin is an exosomal cargo protein. We also present the first evidence that exosomal cargo maspin inhibits migration of recipient fibroblast cells. In light of the overall tumor suppressive effects of epithelial-specific maspin, our results support the development of novel exosomal maspin-based strategies for cancer diagnosis and treatment.

## RESULTS

### Maspin is secreted as both a soluble and an exosomal cargo protein

**Figure 1:**
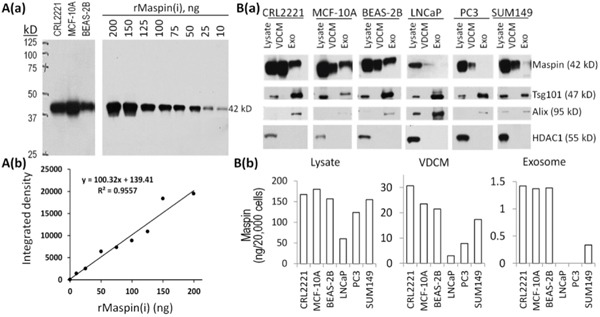
Distribution of maspin in different subcellular compartments by different epithelial cell lines **A**. WB of maspin in the lysates of normal epithelial cell lines (CRL2221, MCF-10A and BEAS-2B) and of rMaspin(i) **a**. rMaspin(i) dose-dependent WB detection based on densitometric analysis using ImageJ software **b**. **B**. Detection and quantification of maspin in the cell lysates, VDCM and exosomes from normal (CRL2221, MCF-10A and BEAS-2B) and tumor cells (LNCaP, PC3 and SUM149). WB detection of maspin. For each cell line, 20 μg of total lysate, 20 μL of VDCM and 20 μL of exosomes were loaded. WB of Tsg101 and Alix were used as markers of exosomes, whereas WB of HDAC1 was used as a marker for the cell lysate fractions **a. b**. Quantification of maspin in each sample based on the densitometric analysis of the maspin bands in B(a). relative to the standard curve in A(b).

To quantitatively assess the distribution of maspin in total cell lysates, vesicle depleted conditioned media (VDCM), and exosome fractions, we first performed western blotting (WB) of purified recombinant maspin produced by baculovirus-infected insect cells, rMaspin(i) [38] and constructed a working dose-response curve based on the linear detection range of 10-200 ng (R^2^= 0.96, Figure 1A). The maspin antibody was highly specific as it only detected the 42 kDa maspin band in the total lysates of three normal epithelial cell lines (CRL2221, MCF-10A and BEAS-2B). Subsequently, the WB detection of maspin in the cell extracts of six different cell lines was quantified based on this working dose-dependent curve (Figure 1B). As compared to the normal epithelial cell lines, human cancer cell lines (LNCaP, PC3, and SUM 149) expressed variable amounts of maspin, with LNCaP cells expressing the lowest level (Figure 1B).

To determine whether epithelial cells spontaneously secrete maspin not only as a soluble protein but also as an exosome-associated protein, and whether the secretion of maspin is further differentially regulated in tumor cells, we prepared VDCM and exosome fractions from the aforementioned normal and tumor cell lines grown in serum free media. For WB of maspin in different fractions (Figure 1B(a)), we loaded 20 μg of total lysate protein, equivalent to the amount of protein derived from approximately 2 × 10^4^ cells; 20 μL of concentrated VDCM, equivalent to the protein secreted by 1.2 × 10^6^ cells; and 20 μL of exosomal suspension, equivalent to the exosomes produced by 1.2 × 10^7^ cells. When quantification of maspin in each fraction was normalized by 2 × 10^4^ cells, (Figure 1B(b)) we found that maspin was predominantly associated with the cell lysates of both normal and tumor cells. The amount of soluble maspin secretion was approximately 1/7-1/5 of the maspin in the corresponding cell lysate, with maspin secretion into VDCM by tumor cell lines being disproportionally lower. Exosomal maspin derived from normal epithelial cells was approximately 1/12 of that in the corresponding cell lysate. However, essentially no maspin was detected in the exosomes of LNCaP and PC3 cells and the exosomal maspin derived from SUM149 cells was at least 50-fold less than that in the corresponding cell lysate. Judging from the WB detection, we successfully eliminated cross-contamination in the VDCM and exosomal fractions. As shown in Figure 1B(a), Tsg101, used as a positive control of exosomal resident molecules [39], was detected in the cell lysate and exosomal fractions, but not in the VDCM fractions. Consistently, the exosome-associated molecule Alix [39] was detected in the lysate and exosomal fractions, but not in VDCM fractions of any cell line. In parallel, HDAC1, a nuclear protein and one of the most abundant cellular proteins [30], was only detected in the cell lysate fractions.

**Figure 2:**
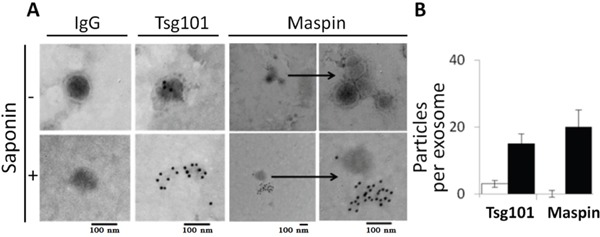
Characterization of exosome-associated maspin under non-permeabilizing (without saponin) and permeabilizing conditions (with saponin) **A**. Representative EM images of immunogold detection of maspin and Tsg101 of MCF-10A-derived exosomes in the presence or absence of saponin. Pre-immune IgG was used as a control. **B**. Quantification of the total number of immunogold particles under the microscope is expressed as the number of particles per exosome. The bars represent the standard errors of three independent repeats.

**Figure 3:**
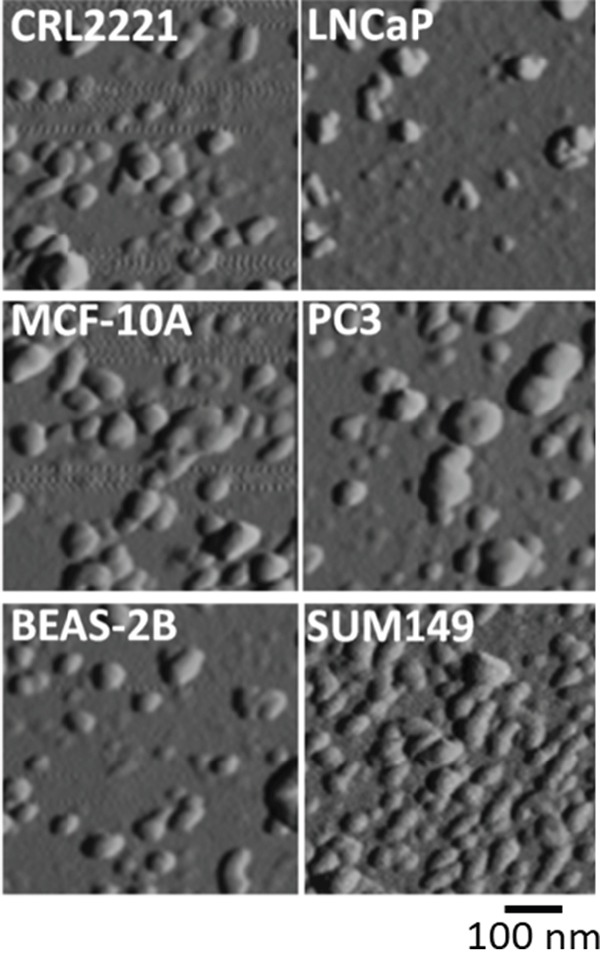
Visualization of exosomes by atomic force microscope (AFM) Representative AFM images of exosomes derived from normal (CRL2221, MCF-10A and BEAS-2B) and cancer (LNCaP, PC3 and SUM149) cell lines.

We verified the properties of our exosomal particles by electron microscopy (EM), a gold standard in the field. Consistent with the general observation [40], exosomes derived from MCF-10A cells featured singular cup-shaped particles of less than 100 nm in diameter with intact continuous bilayer membranes (Figure 2A). To better assess the size distribution of the exosomal particles, we performed atomic force microscopy (AFM), a technique that allows the evaluation of a large number of microvesicles. As shown in Figure 3, exosomes purified from normal epithelial cell lines (CRL2221, MCF-10A and BEAS-2B) and tumor cell lines (LNCaP, PC3 and SUM149) were all in a similar size range and had a high level of membrane integrity. To precisely measure the exosome sizes, dynamic light scattering (DLS) was performed. As summarized in Table 1, normal and tumor-derived exosomes were all 40-90 nm in diameter. The zeta potential of the exosomes was measured using a combination of laser Doppler velocimetry and phased analysis light scattering. As summarized in Table 1, all the exosome particulates, whether from normal or tumor cells, had statistically undistinguishable zeta potentials, ranging from -11 to -13.8 mV.

**Table 1:** Size distribution and surface zeta potential of exosomes

Cell Type	Cell Line	Size (nm) (n)	Zeta Potential (mv) (n)
Normal	CRL2221 MCF-10A BEAS-2B	53.6 ± 12.8 (5) 92.9 ± 42.7 (7) 41.2 ± 8.8 (3)	−13.8 ± 0.67 (3) -12.6 ± 1.85 (3) -13.0 ± 0.91 (3)
Tumor	LNCap PC3 SUM149	55.4 ± 5.3 (4) 87.2 ± 10.1 (3) 91.1 ± 1.8 (3)	−11.2 ± 0.62 (3) -11.0 ± 0.69 (3) -11.7 ± 0.48 (3)

n: number of experimental repeats

To examine whether maspin is encapsulated within the exosomes, immunogold labeling and EM visualization of maspin and Tsg101 in the exosomes of MCF-10A cells were performed in the presence or absence of saponin, a membrane-permeabilizing detergent. The immunogold particles per exosome were enumerated for semi-quantitative analysis (Figure 2B). As expected, the Tsg101 immunogold particles were predominantly detected in permeabilized exosomes, but not on the membrane of non-permeabilized exosomes. In parallel, the maspin immunogold particles had a distribution pattern similar to that of Tsg101 thus demonstrating that maspin is indeed an exosomal cargo protein.

### The dual mechanisms of maspin secretion

**Figure 4:**
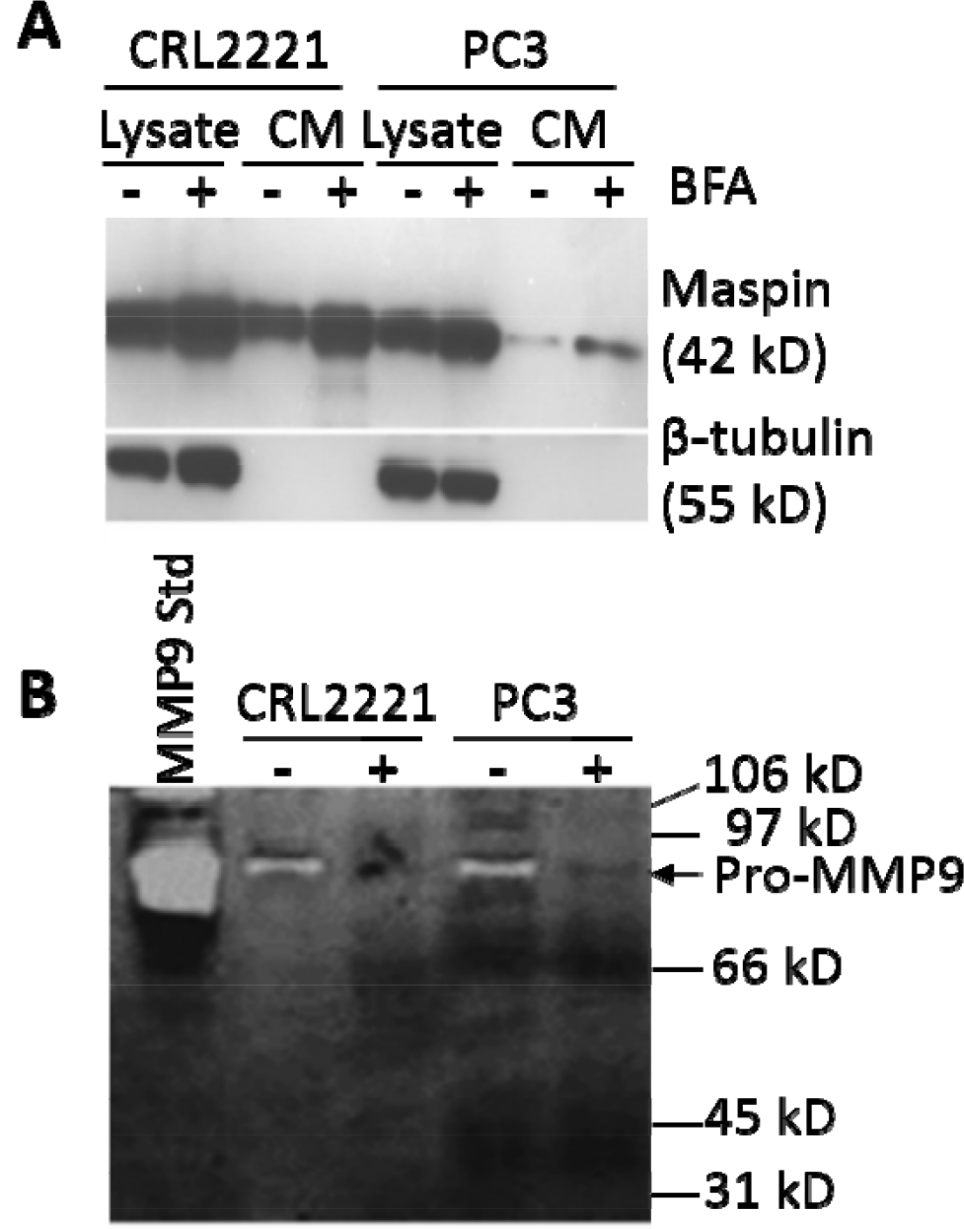
Soluble maspin is secreted by a non-classical secretory pathway **A**. WB of maspin in the lysates and serum-free conditioned media (SFCM) of CLR2221 and PC3 cells. Loading of cell lysate proteins was normalized relative to β-tubulin. The SFCM of each sample was collected from the culture of 6 x10^7^ cells. **B**. Gelatin-based zymographic analysis of gelatinases in the SFCM of CLR2221 and PC3 cells, respectively. Purified pro-MMP9 (10 ng) was loaded as a standard. In (A) and (B), the cells were either untreated or treated with BFA for 24 h.

In addition to the secretion of maspin *via* exosomes, maspin was also secreted as a free protein in the VDCM fractions as expected based on our earlier reports [20, 23]. It is well known that most of the soluble secreted proteins, such as the zymogen form of matrix metalloproteinase 9 (pro-MMP9), depend on their leader sequences to traffic through the classical endoplasmic reticulum (ER)-to-Golgi secretory pathway [41]. Maspin is a leaderless protein, and may not be secreted via the classical secretory pathway [14, 15]. When CRL2221 and PC3 cells were treated with brefeldin A (BFA), an inhibitor of the ER-Golgi secretory pathway [42], the secretion of soluble maspin in VDCM was actually increased (Figure 4A), while the secretion of pro-MMP9 was significantly inhibited, as expected [43] (Figure 4B). It was noted that upon BFA treatment the total level of maspin expression was increased in both CRL2221 and PC3 cell lines. This BFA-induced maspin expression may be a result of non-classical secretory mechanisms that are dependent on the level of maspin expression.

**Figure 5:**
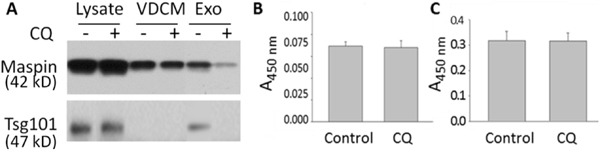
Endosome inhibitor blocks exosomal, but not soluble, maspin secretion **A**. WB of maspin and Tsg101 in the total cell lysates, VCDM and exosomes derived from CRL2221 cells. **B**. Absorbance measurements at 450 nm of the chromogenic activity of LDH that was released to the conditioned media. **C**. Absorbance measurement at 450 nm for the MTT cell viability assay. In (A-C), the cells were either untreated (control) or treated with 5 μM chloroquine (CQ) for 24 h. In (B-C), data represent the average of thee repeats, and the bars represent the standard errors.

The biogenesis and sorting of exosomes depend on the endosomes. To test whether the exosomal secretion of maspin is sensitive to endosome inhibitors, CRL2221 cells were treated with chloroquine (CQ) which disrupts exosomal secretion by raising the pH of the endosome lumen [44–46]. While some reported that CQ treatment leads to increased exosome secretion [47, 48], others showed that CQ can actually inhibit exosome secretion [49]. In our study, as shown in Figure 5A, CQ treatment significantly reduced the level of exosome-associated maspin and almost completely abolished the secretion of exosome-associated Tsg101. In parallel, the levels of maspin and Tsg101 in the lysate and VDCM fractions were not affected by the same treatment. As judged by the lack of leakage of intracellular lactate dehydrogenase (LDH) into VDCM (Figure 5B) and the absence of cell viability loss (Figure 5C), the detected extracellular maspin, either in the VDCM fraction or exosomes, was not likely a result of spontaneous or induced cell lysis. These data suggest that the regulation of exosomal maspin secretion may be distinct as compared to that of soluble maspin secretion, although endosomes may be involved in both pathways. It is noted that, compared to the level of exosomal maspin collected from cell cultures over a 3 day period (Figures 1 & 2), the level of exosomal maspin from a 24-hour culture of CRL2221 cells was significantly lower. A similar parallel reduction of exosomal Tsg101 was observed. These results demonstrate the time-dependence of exosomal maspin secretion.

### Exosome-associated maspin is a paracrine factor

**Figure 6:**
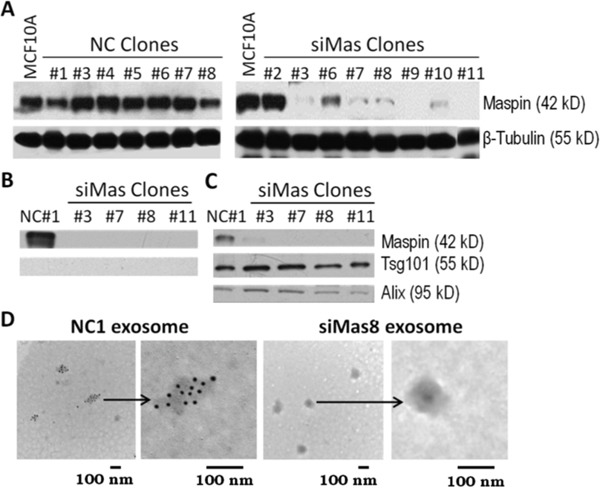
Reduction of maspin secretion by maspin knockdown in MCF-10A cells **A**. WB of maspin and β-tubulin in the lysates of cells stably transfected by a noncoding shRNA construct (NC) and a maspin-specific shRNA construct (siMas), respectively. **B**. WB of maspin and Tsg101 in the VDCM of the stably transfected clonal cell lines. **C**. WB of maspin, Tsg101 and Alix in the exosomes derived from the indicated transfected clones. **D**. Representative EM images of immunogold labeled maspin in exosomes derived from NC1 and siMas8 cell lines, respectively, under permeabilizing conditions.

Considering that exosomal cargo proteins may be delivered in a paracrine manner to heterotypic recipient cells, it is important to investigate the biological effects of exosome-associated maspin. To generate exosomes which are distinct only at the level of maspin cargo protein, we stably transfected MCF-10A cells with either a short hairpin RNA of a nonspecific sequence (designated as NC) or maspin shRNA (designated as siMas). The stably transfected clones, selected based on antibiotic resistance, were screened by WB for the level of maspin expression. As shown in Figure 6A, while the NC shRNA did not alter the overall maspin expression in the NC clones, all siMas clones, with the exception of clone #2, expressed maspin at reduced levels as compared to that in the parental cell line. As expected, intracellular β-tubulin protein, which is not homologous to maspin at the mRNA and protein levels, remained unchanged in the lysates of both NC and siMas clonal cell lines. Interestingly, the reduction of maspin in the VDCM (Figure 6B) and exosomal (Figure 6C) fractions of the siMas clones seemed to be disproportional as compared to the reduction in the corresponding lysate fractions (Figure 6A). The absence of Tsg101 in the VDCM fractions confirmed the lack of contamination by exosomal or lysate fractions. Conversely, the uniform detection of Tsg101 and Alix in the exosome fractions further demonstrates the specificity of maspin knockdown in the siMas clones. To ascertain the maspin knockdown in the exosomes by siMas clonal cell lines, we performed immunogold labeling and EM under permeabilizing conditions. As shown in Figure 6D, immunogold detection was highly specific with negligible nonspecific background. Importantly, immunogold particles were only detected in the exosomes derived from NC1 cells, but not in the exosomes derived from siMas8 cells.

**Figure 7:**
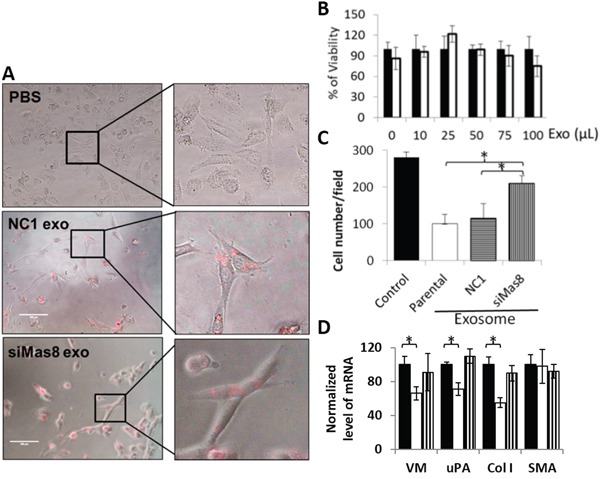
Effect of exosomal maspin on the chemotaxis of recipient fibroblast cells **A**. Representative composite image of fluorescence and phase contrast microscopies of NIH3T3 cells treated with PKH26-labeled exosomes, 20x. Cells treated with dye in PBS were used as a negative control. **B**. The MTT viability assay of NIH3T3 cells treated with either NC1 (■) or siMas8 (□) derived exosomes at the indicated volumes. Data were normalized by the results of NC1-exosome treated cells, and represent the average of thee repeats. The bars represent the standard errors. **C**. Chemotaxis of NIH3T3 cells treated with PBS only without exosomes (negative control), the parental MCF-10A-derived, NC1-derived or siMas8-derived exosomes. Cell migration is presented as the average number of cells per microscopic field. The bars represent the standard errors. *p< 0.001. **D**. Quantification of mRNA expression by real-time PCR in untreated control (■), NC1 exosome-treated (□), and siMas8 exosome-treated cells (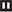). The expression level was based on the ΔΔ Ct formula, and presented as an average relative to the corresponding control. In (C) and (D), * indicates a statistically significant difference of p < 0.01.

Intracellular maspin was shown to specifically inhibit HDAC1 and consequently to reprogram tumor cells gene expression profile towards a less motile and invasive phenotype [5, 6]. Earlier, we showed that maspin expression in tumor cells also reduced the reactivity of tumor stroma [6, 8]. To test whether exosome-delivered maspin exerts a biological effect by inhibiting motility and chemotaxis of stromal recipient cells, we used NIH3T3 fibroblast cells as an experimental model. It has been reported that exosomes can be labeled with red fluorescent cell linkers PKH26 [50] or PKH67 [51] diluted in Diluent C, a proprietary aqueous solution designed to maintain cell viability, while maximizing dye solubility and staining efficiency, to allow for the monitoring of exosomal uptake by live cells. To determine the efficiency of exosome uptake by NIH3T3 cells we used PKH26 to label exosomes derived from NC1 and siMas clones, respectively. To validate exosome specific labeling, the fluorescence of exosomes treated with PKH26 in PBS only was also examined as a negative control. As shown by the composite image of fluorescent and phase contrast microscopy, PKH26 was only detected inside the exosome-treated NIH3T3 cells, but not elsewhere in the culture dish (Figure 7A). Further, the PKH26 labeled NC1 exosomes and the siMas exosomes were taken up by NIH3T3 fibroblast cells with similar efficiencies. It is important to point out that the PKH26 labeling of the exosomal membrane is not through covalent bonds; therefore, PKH26 may dissociate from exosomes upon internalization. Thus, internalized PKH26 may not specifically mark the subcellular localization of the exosome-delivered maspin (and other exosome cargo proteins).

As shown in Figure 7B, the growth and viability of NIH3T3 cells were not affected by either NC1 exosomes or siMas exosomes. Interestingly, the exosomes isolated from parental MCF-10A or NC1 cells inhibited motility and chemotaxis of NIH3T3 cells. However, this inhibitory effect was greatly diminished by the exosomes derived from the siMas8 cells (Figure 7C), suggesting that the down-regulation of maspin in epithelial-derived exosomes may unleash the tumor-associated stromal reactivities. Based on the evidence that intracellular maspin regulates gene transcription by inhibiting HDAC1, we tested the possibility that maspin delivered *via* exosomes into fibroblast cells may reverse the transcription control by HDAC1. As shown in Figure 7D, expression of vimentin (VM), uPA and collagen 1 (Col 1) by the NIH3T3 cells exposed to NC1 exosomes was significantly down-regulated at the mRNA level relative to cells untreated or treated with siMas8 exosomes. Since similar results were obtained when prostate tumor cells were stable transfected to re-express maspin [6], these results suggest that maspin, whether exosomal or endogenously expressed, exerts similar effects in the cells gene expression profile. Of note, VM and uPA have been shown to support cell motility and invasion, while Col I upregulation is common in inflammatory stromal response. As an epithelial-specific protein, it is not surprising that maspin did not affect the expression of smooth muscle actin (SMA), which is predominantly expressed by smooth muscle cells and is an established marker of myofibroblast formation [52]. Taken together, this is the first functional evidence that exosomal cargo maspin of epithelial origin may act as a paracrine factor to block the migratory activity of fibroblasts. Since tumor associated fibroblasts may become activated with increased chemotaxis and promote tumor progression [53], our data suggest that epithelial derived exosomal maspin may suppress tumor-induced reactive stroma, at least in part, by inhibiting HDAC1-dependent transcriptome.

## DISCUSSION

Earlier, it was reported that maspin is secreted as an exosomal protein by irradiated tumor cells in a p53-dependent manner [25]. In this report we provided the first evidence that human epithelial cell lines naturally secrete maspin as an exosomal cargo protein. While maspin was abundantly present in normal cell-derived exosomes, it is disproportionally reduced in tumor cell-derived exosomes, possibly due to differential partitioning of maspin in normal vs. tumor cells. Of note, all three normal epithelial cell lines used in this study express wild type p53, whereas each of the three tumor cell lines carries mutated p53 [54, 55]. While it is possible that wild type p53 may be involved in the spontaneous exosomal maspin secretion, it remains unclear whether mutated p53 was responsible for the significant reduction in exosomal maspin secretion by tumor cells. Nonetheless, since it has been shown that tumor cell-derived exosomes typically carry oncogenic cargo molecules, our new data suggest that tumor cell-derived exosomes may carry less tumor suppressor molecules such as maspin. Indeed, loss of maspin in normal cell-derived exosome cargo may significantly elevate stimulatory effects on fibroblast chemotaxis (Figure 7).

Consistent with earlier reports, maspin was predominantly an intracellular protein, but was also secreted as a soluble protein into the cell culture media. Until recently it was widely accepted that proteins required an N-terminal signal peptide to be recognized and trafficked through the classical ER-Golgi network for optimal folding and post-translational processing, and to be secreted into the extracellular milieu. However, it is now recognized that leaderless proteins, such as interleukin 1β (IL-1 β), can be secreted *via* multiple non-classical pathways including endolysosomal exocytosis and exosomes [56–58]. Likewise maspin does not contain a leader sequence for the classical ER-Golgi secretion mechanism. Based on a neutral network (NN) score of 0.5 assigned to maspin by the SecretomeP program (http://www.cbs.dtu.dk/services/secretomeP/), maspin is predicted to be secreted by a non-classical pathway [59]. Consistently, BFA, an inhibitor of the classical secretion pathway, had no effect on soluble maspin secretion (Figure 4).

It remains unclear how cells coordinate the secretion of soluble maspin protein and maspin-containing exosomes. Although the mechanisms of unconventional protein secretion are still evolving [60] three mechanisms have been generally implicated in non-classical secretion, namely secretion though specific plasma membrane anchorage and subsequent exocytosis, specific plasma membrane transporters, and trafficking through endosome sub-compartments (MVB) [61]. Interestingly, among all its serpin homologues and orthologues, human maspin is the only protein with an intramolecular KDEL motif C-terminal to its RCL. It has been shown that when KDEL sequence is located at the very C-terminus, it renders the protein a resident of the ER [62]. We have shown that a conservative point mutation of KDEL to KEEL leads to the exclusive nuclear localization of maspin [18]. Based on the report of Johannes *et al*. [63], an intramolecular KDEL sequence may be subject to glycosylation which subsequently directs retrograde protein transport *via* endosomes. While it remains to be determined whether the KDEL sequence of maspin plays a role in the dual mechanisms of maspin secretion, we tested whether MVB was a rheostat of the dual maspin secretion pathways. We treated the cells with CQ, which is known to inhibit the exosomal secretory pathway by inducing phospholipidosis [64]. Under our experimental conditions, CQ effectively eliminated the secretion of exosomes along with maspin. However, it did not affect the secretion of soluble maspin (Figure 5). These data suggest that the secretion of exosomal maspin and the secretion of soluble maspin may proceed concomitantly, to different extents, through independent pathways.

The full benefit of the dual mechanisms of maspin secretion needs to be further investigated. While both forms of maspin may contribute to the homeostasis of differentiated epithelia, a conceivable difference between soluble maspin in the extracellular space and the exosome-encapsulated maspin is where it exerts its biological function. Extracellular maspin was shown to bind and inhibit the proteolytic activation of pro-uPA that is associated with its cell surface-anchored receptor urokinase receptor (uPAR) [20, 24]. The molecular interaction of maspin with the uPAR/pro-uPA complex also quenches the uPA proteolytic cascade by triggering low-density lipoprotein receptor (LRP)-mediated internalization, which helps explain the tumor suppressive activities of either secreted maspin or purified maspin protein in blocking tumor cell detachment, motility, invasion and tumor-induced angiogenesis [8, 10, 11, 23, 24]. The underlying mechanism of internalization of soluble maspin may include the perturbation of Rac1 signaling [65, 66].

Earlier, it was shown that maspin expression in tumor cells reverted the cells to an epithelial-like phenotype due to the reprogramming of a small subset of HDAC1 target genes [6]. Similarly, in the current study, we showed that maspin-containing exosomes delivered to fibroblasts significantly decreased the expression of the mesenchymal marker VM [67], uPA and Col 1. Interestingly, the internalized recombinant maspin was shown to down-regulate the level of uPA mRNA [23]. We speculate that intact maspin delivered into recipient cells by exosomes may simulate endogenously expressed intracellular maspin in its subcellular localization and biological functions and may not involve LRP and Rac1. However, it is likely that both internalized maspin protein and exosome-delivered maspin protein are intact and can likely function as an endogenous intracellular HDAC1 inhibitor. To this end, data of the current study raise an intriguing possibility that epithelial-derived maspin may be directly trafficked to mesenchymal cells to extend the tumor suppressive influence of epithelial cells on the stroma.

The overall maspin expression has been reported to correlate with a better prognosis in the clinic for primary tumors in a variety of organs including prostate [68] and breast [69]. However, the opposite appears to be true for ovarian cancer patients. We believe that this discrepancy can be ameliorated if maspin subcellular localization, rather than the overall level of maspin expression, is correlated with tumor grade. We have demonstrated this to be the case for non-small cell lung (NSCL) adenocarcinoma. Maspin nuclear localization in histopathological samples of extracted NSCL adenocarcinomas was found to correlate with better patient prognosis [3, 70, 71]. In light of the evidence from the current study, when correlating maspin expression with patients’ prognosis, we also need to take into consideration secreted soluble and exosomal maspin in order to accurately determine the state of epithelial differentiation of the tumor cells as well as changes in tumor stroma.

Therapeutic application of maspin may be effective in treating invasive and metastatic tumors. To this end, to restore maspin expression specifically in tumor cells seems challenging since the transcriptional control of gene expression is complex and plastic. An alternative approach is to deliver biologically active maspin to target cell populations. Earlier, we have shown that soluble maspin suppresses tumor cell invasion and motility, and has a half-life of approximately 12 h [19, 38]. The internalized maspin may be subsequently degraded in a lysosome-dependent manner [23]. Considering the strong rationale for exosome-mimicking drug delivery strategies, evidence from the current study raises the intriguing possibility that pharmacological maspin delivery by exosomes may be used for cancer treatment in the future.

## MATERIALS AND METHODS

### Cell lines, cell culture media and reagents

The normal immortalized lung epithelial cell line BEAS-2B was a gift from Dr. Fulvio Lonardo (WSU SOM, Detroit, MI) [72]. The spontaneously immortalized human breast epithelial cell line MCF-10A [73] was a gift from Dr. Fred Miller (WSU SOM, Detroit, MI). The human primary inflammatory breast cancer cell line SUM149 was a gift from Dr. Stephen Ethier (Medical University of South Carolina, Charleston, SC) [74]. The normal immortalized human prostate epithelial cell line CRL2221, human prostate carcinoma cell lines PC3 and LNCaP, and mouse fibroblast cell line NIH3T3 were from the American Type Culture Collection (Manassas, VA).

LNCaP and PC3 cells were maintained in RPMI 1640 medium supplemented with 5% (v/v) fetal bovine serum (FBS), penicillin (100 U/mL), streptomycin (100 μg/mL), L-Glu (2 mM), Hepes (10 mM), NaHCO_3_ (1.5 mg/mL) and non-essential amino acids (NEAA, 1 mM). CRL2221 cells were maintained in keratinocyte serum-free medium (KSFM) supplemented with penicillin (100 U/mL) and streptomycin (100 μg/mL). The MCF-10A and NIH3T3 cells were maintained in DMEM/F-12 medium supplemented with 5% (v/v) FBS, L-Glu (2 mM), penicillin (100 U/mL), streptomycin (100 μg/mL), amphotericin B (0.5 μg/mL), cholera toxin (100 ng/mL), hydrocortisone (HC, 1 μg/mL), epidermal growth factor (EGF, 10 ng/mL), and insulin (5 μg/mL). SUM149 cells were maintained in Ham's F-12 medium supplemented with 10% (v/v) FBS, penicillin (100 U/mL), streptomycin (100 μg/mL), insulin (5 μg/mL), and HC (1 μg/mL). BEAS-2B cells were maintained in LHC-8 medium supplemented with penicillin (100 U/mL) and streptomycin (100 μg/mL). All cells were cultured in a humidified incubator at 37°C with 5% CO_2_.

All cell culture media, L-Glu, penicillin, streptomycin, Hepes, NEAA, and EGF were purchased from Life Technologies (Gaithersburg, MD). Puromycin, cholera toxin, insulin, amphotericin B, BFA, chloroquine (CQ), and all inorganic salts (biological grade with the highest level of purity) were purchased from Sigma-Aldrich (St. Louis, MO). Mouse monoclonal antibody against maspin was purchased from BD Biosciences (#554292, BD Pharmingen, San Jose, CA). Mouse monoclonal antibody against HDAC1 was purchased from Millipore (#06720, Millipore, Grand Island, NY). Mouse monoclonal antibodies against Tsg101 (ab83, clone 4A10) and Alix (ab117600), and rabbit polyclonal antibody against β-tubulin (ab6046) were purchased from Abcam (Cambridge, MA). Anti-mouse secondary antibody and anti-rabbit polyclonal secondary antibody were purchased from GE Healthcare (Buckinghamshire, UK).

### Preparation of vesicle-depleted conditioned media (VDCM) and exosomes

Cells were cultured in maintenance media for 3 days to reach approximately 70% confluence. The conditioned media (CM) was collected and centrifuged at 16,000 x g for 30 min at 4°C to deplete high molecular weight microvesicles. The resulting supernatant was centrifuged at 100,000 x g for 24 h at 4°C to pellet the exosomes. The supernatant, the vesicle-depleted CM (VDCM), was concentrated to 1000 μL using the Thermo Scientific Pierce concentrator for subsequent analysis. The exosomes were washed by two cycles of re-suspension in phosphate buffered saline (PBS) and centrifugation at 200,000 x g for two hours at 4°C. The pellet was resuspended in 100 μL of PBS.

### Atomic Force Microscopy (AFM)

To prepare cleaved mica for exosome immobilization and AFM detection, each freshly cleaved grade 5 muscovite mica (1 cm x 1 cm) was treated with 50 μL of 10 mM MnCl_2_ for 30 s, and blow-dried with filtered compressed air. Exosomal particles resuspended in 20 μL of PBS were loaded on the MnCl_2_-treated cleaved mica, incubated for 2 min and dried with filtered compressed air. To image the exosomes using a Multimode IIIa (Digital Instruments) and a Dimension 3100 (VEECO, Bruker, Billerica, MA), tapping mode in air was performed using silicon probes (Vistaprobe; Phoenix, AZ) with a nominal radius of curvature of 10 nm and cantilever spring constant of 48 N/m as recommended by the manufacturer. The surface was imaged continuously at an average rate of 1−2 Hz on an area of 1×1−5×5 μm^2^. The ranges of frequency, amplitude, integral, and proportional gains used were 7.5-8.5 kHz, 0.5−1 V, 0.5−2 CU, and 0.75−3 CU, respectively. All AFM images were analyzed using the software package Nanoscope version 5.12b (VEECO, Plainview, NY).

### Dynamic Light Scattering (DLS) for size distribution and zeta potential

The size of the exosomes was measured by dynamic light scattering with the Nanosizer ZS (Malvern Instruments, Worcestershire, UK). Briefly, exosomes were resuspended in 50 μL of PBS and transferred to a microcuvette (ZEN0040, Malvern Instruments) [75]. The backscattering angle Θ was fixed at 172° with a laser wavelength λ=633 nm. The size of the exosomal particles was calculated as hydrodynamic diameters (DH) based on the Stokes-Einstein equation: DH=kT/3πηD, where k is the Boltzmann constant, T is the absolute temperature, η is the viscosity and D is the diffusion coefficient. The values of D were obtained from autocorrelation function via the cumulate fitting. The DH range was 1 nm to 6 μm. To measure the surface charge, exosomes were resuspended in 1 mL of PBS. The Zeta potential of the exosomes was measured with a combination of laser Doppler velocimetry and phase analysis light scattering in a disposable capillary cell (DTS1070, Malvern Instruments).

### Electron Microscopy (EM)

Exosomes were re-suspended in 4% (v/v) paraformaldehyde in 0.1 M sodium phosphate buffer (pH 8), and incubated for 24 h. Then, 5 μL of exosomes were allowed to adsorb onto carbon-coated, 400 mesh, nickel grids (Electron Microscopy Sciences, Hatfield, PA). The exosomes were incubated in 0.3% saponin diluted in PBS for permibialization. Exosomes were washed once with PBS and twice with glycine buffer. The exosomes were then incubated in blocking buffer (1% (w/v) cold-water fish gelatin in PBS) for 45 min. For primary antibody labeling, exosomes were incubated for 1 h in 1:10 dilution of maspin antibody, Tsg101 antibody or mouse IgG. After washing with 0.1% cold-water fish gelatin six times, exosomes were incubated in a 1:10 dilution of conjugated gold mouse secondary antibody for 30 min. The exosomes were washed six times in PBS followed by incubation in 1% (v/v) glutaraldehyde in 0.1 M phosphate buffer for 30 min. The exosomes were then washed in ddH2O. Negative staining was conducted in 2% uranyl oxalate (pH 7.0) for 5 min. The exosomes were quickly rinsed once in ddH2O and allowed to air dry. Exosomes were visualized using the JEOL 2010 FasTEM instrument at 200 kV located at the John Dingell Veteran Hospital (Detroit, MI).

### Maspin knockdown by stable transfection

MCF-10A cells were transfected with the pGIPZshRNA-mir lentiviral plasmids (Thermo Scientific, Asheville, NC) according to the manufacturer's instructions. Briefly, MCF-10A cells were seeded in 100 mm cell culture plates. At 50% confluence, cells were transfected with either a mixture of maspin shRNA plasmids (RHS4430-98895314, RHS4430-99297939, RHS4430-99139485) or the noncoding shRNA plasmid (RHS4346) using the X-treme GENE 9 DNA transfection reagent (Roche Applied Science, Indianapolis, IN). Stable transfected clones, selected based on resistance to 10 μg/mL puromycin, were maintained in DMEM/F-12 medium containing 5% fetal calf serum and 5 μg/mL puromycin.

### Tracking exosome uptake

Exosomes were washed once with PBS and labeled with the PKH26 Red Fluorescent dye using a Cell Linker Kit (Sigma Aldrich) according to the manufacturer's instructions. Briefly, exosomes were resuspended in 1 mL Diluent C (or PBS) and 4 μL PKH26 was mixed with 1 mL Diluent C (or PBS) separately. The exosome suspension and the PKH26 solution were mixed and incubated for 4 min. The labeling reaction was stopped by adding an equal volume of 1% BSA. The labeled exosomes were ultra-centrifuged at 100,000 ×g for 2 h, washed with PBS, and ultra-centrifuged again at 100,000 ×g for 2 h and then finally re-suspended in 1 mL of defined KSFM. The labeled exosomes were added to NIH3T3 mouse fibroblast cells that had been cultured in Petri dish for 24 h. Live cell imaging of NIH3T3 cells after exosome treatment was performed using the Model DM IRB Leica fluorescence microscope (Buffalo Grove, IL).

### Chemotaxis assay

NIH3T3 cells were seeded in 6 well plates in maintenance media and cultured for 24 h before exosomes in PBS were added at the dilution of 1:100. Exosome treated cells were collected after 24 h and seeded into the upper chambers of Corning transwell plates (Sigma-Aldrich) in serum-free DMEM/F-12 medium. Cells treated with PBS were used as a negative control. The cells that migrated to the bottom side of the chamber were stained and counted under the microscope as previously described [24].

### Cell viability and plasma membrane integrity assays

Cells were seeded in 96-well plates at the density of 20,000 cells/mL. Cell viability was assessed with the WST-1 Reagent (Roche Diagnostics, San Francisco, CA) according to the manufacturer's instructions. To measure the extent of plasma membrane leakage, the activity of intracellular enzyme lactate dehydrogenase (LDH) was measured in the CM using the LDH kit (Cayman Chemical, Ann Arbor, MI), according to the manufacturer's instructions.

### RNA extraction and mRNA quantification by real time PCR (q-RT-PCR)

The RNA from exosome treated NIH3T3 mouse fibroblasts was extracted (RNeasy Mini kit, Qiagen, Valencia, CA) and reverse-transcribed (iScript cDNA synthesis kit, Bio-Rad, Hercules, CA). Q-RT-PCR was performed as described [76] using a iQ™5 Multicolor Real-Time PCR Detection System. The sequences of the primers are listed in Supplementary Table 1. Normalization of q-RT-PCR results was performed using the ΔΔCt method [77].

### Miscellaneous methods

For cell lysate preparation, cells were washed with PBS, detached with 0.25% trypsin (Life Technologies, Gaithersburg, MD), re-suspended in PBS and centrifuged at 2,000 x g for 5 min. Cells were lysed with cold RIPA lysis buffer and centrifuged at 16,000 x g for 30 min at 4°C [22]. The supernatant was collected as the total cell lysate. Protein concentration measurements were carried out using the Pierce BCA Protein Assay Reagent Kit (Rockford, IL). Western blotting (WB) was done as previously described [22]. Densitometric quantification of the WB protein detection was performed using the ImageJ program, a public domain image processing software developed at the National Institutes of Health (https://imagej.nih.gov). Briefly, a WB scanned film image (in TIFF format) was imported into ImageJ, the lanes of interest were selected, and lane profile plots were generated. Lines were drawn to enclose the peaks of interest and the peak areas were integrated and converted into pixel intensities. For statistical analysis, one way analysis of variance (ANOVA) was performed using the SigmaPlot software (Chicago, IL).

## SUPPLEMENTARY MATERIALS TABLE



## Abbreviations

AFM: atomic force microscopy
BFA: brefeldin A
CQ: chloroquine
CM: conditioned media
DLS: dynamic light scattering
EM: electron microscopy
EGF: epidermal growth factor
ESCRT: endosomal sorting complex
FBS: fetal bovine serum
HDAC1: histone deacetylase 1
KSFM: keratinocyte serum-free medium
L-Glu: L-glutamine
LDH: lactate dehydrogenase
LDL: low-density lipoprotein
MMP-9: matrix metalloproteinase 9
MVB: multivesicular body
NEAA: non-essential amino acids
uPA: urokinase type plasminogen activator
uPAR: urokinase type plasminogen activator receptor
VDCM: vesicle-depleted conditioned media
SFCM: serum-free conditioned media WSU SOM, Wayne State University School of Medicine
