# Supplementary material for: The secretion and biological function of tumor suppressor maspin as an exosome cargo protein
Source: Oncotarget. 2016 Nov 11;8(5):8043–56. doi: 10.18632/oncotarget.13302 (PMC5352381; doi:10.18632/oncotarget.13302)
Supplement: Supplementary file 1 [file oncotarget-08-8043-s001.pdf]

The secretion and biological function of tumor suppressor maspin as an exosome cargo protein

SUPPLEMENTARY TABLE

Supplementary Table 1: Sequences of Real-Time PCR Primers

Gene	Primer Sequence
Mouse Maspin	Forward: 5'-GCCGCTCGAGTCGCACAGCTTTCACTG-3'
	Reverse: 5'-CGCGCGAAGCTTGCGTAATTCAATAAT-3'
Mouse uPA	Forward: 5'-CATCCATCCAGTCCTTGCGT-3'
	Reverse: 5'-ACGCATACACCTCCGTTCTG-3'
Mouse α1-SMA	Forward: 5'-GTACCCAGGCATTGCTGACA-3'
	Reverse: 5'-GAGGCGCTGATCCACAAAAC-3'
Mouse Vim	Forward: 5'-GCTCCTACGATTCACAGCCA-3'
	Reverse: 5'-CGTGTGGACGTGGTCACATA-3'
Collagen 1	Forward: 5'-ATCAACCGGAGGAATTTCCGT-3'
	Reverse: 5'-CACCAGGACGACCAGGTTTTC-3'
Actin	Forward: 5'-CCTGACTGAGCGTGGCTATT-3'
	Reverse: 5'-GATGAAGGATGGCTGAACA-3'
Mouse GAPDH	Forward: 5'-AGGTCGGTGTGAACGGATTTG-3'
	Reverse: 5'-TGTAGACCATGTAGTTGAGGTCA-3'
